# HLA-A*11:01 is associated with levetiracetam-induced psychiatric adverse events

**DOI:** 10.1371/journal.pone.0200812

**Published:** 2018-07-18

**Authors:** Tae-Won Yang, Jangsup Moon, Tae-Joon Kim, Jin-Sun Jun, Jung-Ah Lim, Soon-Tae Lee, Keun-Hwa Jung, Kyung-Il Park, Ki-Young Jung, Kon Chu, Sang Kun Lee

**Affiliations:** 1 Department of Neurology, Gyeongsang National University Changwon Hospital, Gyeongsang National University College of Medicine, Jinju, Republic of Korea; 2 Department of Neurology, Laboratory for Neurotherapeutics, Comprehensive Epilepsy Center, Biomedical Research Institute, Seoul National University Hospital, Seoul, Republic of Korea; 3 Program in Neuroscience, Seoul National University College of Medicine, Seoul, Republic of Korea; 4 Department of Neurosurgery, Seoul National University Hospital, Seoul, Republic of Korea; 5 Department of Neurology, Kyungpook National University Chilgok Hospital, Kyungpook National University School of Medicine, Daegu, Republic of Korea; 6 Department of Neurology, Kangnam Sacred Heart Hospital, Hallym University College of Medicine, Seoul, Republic of Korea; 7 Department of Neurology, Seoul National University Hospital Healthcare System Gangnam Center, Seoul, Republic of Korea; University of Catanzaro, ITALY

## Abstract

Levetiracetam (LEV) is effective for focal and generalized epilepsy and is used worldwide because of its relatively few drug interactions and favorable tolerability. However, some psychiatric adverse events (PAEs) have been reported, resulting in drug withdrawal. The pathophysiology of LEV-induced PAE has not yet been elucidated. In this study, we investigated the relationship between PAEs and human leukocyte antigen (HLA) genes. Eleven epilepsy patients, who developed PAEs after the administration of LEV and spontaneously improved after drug withdrawal, were enrolled retrospectively. Genomic DNA from the peripheral blood was extracted, and four-digit allele genotyping of HLA genes was performed. The genotype frequencies of HLA genes were compared to those of 80 patients in which LEV was well tolerated, as well as to 485 individuals from the general Korean population. The frequency of the HLA-A*1101 allele was significantly higher in the LEV-induced PAEs group compared to both the LEV-tolerant group (p = 0.021, OR 4.80, 95% CI 1.30–17.74) and the general Korean population (p = 0.015, OR 4.62, 95% CI 1.38–15.45). This study is the first attempt at investigating the relationship between the HLA system and LEV-induced PAE. The results of this study suggest that the HLA-A*1101 allele could be a risk factor for the development of PAEs.

## Introduction

Levetiracetam (LEV) is a broad-spectrum antiepileptic drug (AED)[1–3], which is used worldwide due to its relatively few drug interactions and favorable tolerability.[4, 5] However, psychiatric adverse events (PAEs) of LEV have been frequently reported, including psychosis, aggression, hostility, irritability, and nervousness.[6, 7] LEV may also be associated with an increased risk of suicidal ideation or behavior, as has been reported in 0.5–0.7% of patients receiving LEV.[6] Up to 7% of the patients discontinue LEV because of the PAEs; additionally, PAEs are the most common reason for withdrawal.[7]

The known risk factors for PAEs during LEV treatment are previous psychiatric history, family psychiatric history and history of febrile convulsions.[7, 8] However, the genetic risk factors for PAEs of LEV have not yet been fully investigated.

Many studies have demonstrated the associations of the human leukocyte antigen (HLA) in patients with various psychiatric diseases, including schizophrenia, schizoaffective disease, and mood disorders.[9–11] Since there is growing evidence that psychiatric diseases are influenced by the immune system in the brain,[12, 13] we hypothesized that PAEs of LEV may be induced by immune-mediated mechanisms related to the HLA system. The HLA associations with adverse events of AEDs are well known, especially in the cutaneous adverse drug reactions.[14–16]

In the current study, we attempted to investigate the HLA-related risk factors for PAEs of LEV for the first time.

## Materials and methods

### Patients

We retrospectively enrolled 11 patient who experienced significant psychiatric symptoms after the administration of LEV among the patients with epilepsy who were treated with LEV at Seoul National University Hospital. A diagnosis of LEV-induced PAE was made when psychiatric symptoms or behavioral changes occurred after administration of LEV and when those symptoms were improved spontaneously after drug withdrawal. Psychiatric symptoms included hallucinations, delusions and behavioral changes consisting of aggression, irritability, and nervousness. When psychiatric symptoms were clinically confirmed, LEV administration was discontinued immediately to prevent serious adverse events. Patients with the following conditions were excluded: (1) previous history of mental illness, (2) severe mental retardation, (3) incomplete clinical data, or (4) uncertainty of LEV as the causative drug. Eighty epilepsy patients who were tolerant to LEV (LEV-tolerant group) were included as a control group. The LEV-tolerant group consisted of patients who did not exhibit any psychiatric symptoms while taking over 2000 mg/day of LEV. Additionally, we used 485 individuals from the general Korean population as the other control group.[17] This study was approved by the Institutional Review Board of Seoul National University Hospital, and written informed consent was obtained from all participants.

### HLA genotyping

After genomic DNA extraction from the peripheral blood of all patients belonging to the LEV-induced PAEs group (LEV-PAEs group) and the LEV-tolerant group, HLA genotyping was performed. Four-digit allele genotyping of the HLA class I and class II genes, including HLA-A, HLA-B, HLA-C, HLA-DRB1 and HLA-DQB1, was performed using direct DNA sequence analysis, according to the established protocols (Biowithus, Seoul, Korea).[15, 16, 18] The frequencies of the abovementioned HLA genes in the general Korean population, which were documented in the previous report, were used as a control group.[17]

### In silico docking

In silico docking was performed as previously described,[16] using the LEV molecule and HLA subtypes, which were observed in the present study. In brief, we obtained the three-dimensional structure of LEV from the Human Metabolome Database (http://www.hmdb.ca) and used a computational program, Autodock Vina,[19] to calculate the docking score of LEV into the HLA molecules. For comparison, docking runs were performed with HLA-A alleles, of which structures are available in the Protein Data Bank database.

### Statistical analysis

The statistical analysis was performed using SPSS Statistics for Windows, version 22.0 (SPSS Inc., Chicago, Ill., USA). Fisher’s exact test was used to identify differences in the HLA frequencies among the following three groups: the LEV-PAEs group, the LEV-tolerant group and the general Korean population. Odds ratios (ORs) and 95% confidence intervals (CIs) were obtained. A two-tailed p-value < 0.05 was considered statistically significant.

## Results

### Clinical characteristics

A total of 11 patients (9 male, 2 female) who experienced LEV-induced PAEs and 80 patients who tolerated LEV treatment were included in this study. Demographics and clinical characteristics of the patients with LEV-induced PAEs are shown in Table 1. Furthermore, types of PAE and LEV dosages at the point of the PAE occurrence are shown in Table 2. Of the 11 patients in the LEV-PAEs group, 7 (63.6%) had aggressive behaviors (aggression and irritability), 3 (27.3%) had psychosis (auditory hallucination in 3 and delusion in 1), and 3 (27.3%) had nervousness. The median dose of LEV was 1000 mg/day (range 250–2000 mg/day) when the PAEs appeared. The majority of the patients (9 of 11, 81.8%) encountered PAEs on a dose less than 1500 mg per day of LEV. All 80 patients in the control group were taking more than 2000 mg/day.

**Table 1:** Demographics and clinical characteristics of the patients with levetiracetam-induced psychiatric adverse events.

No	Sex	Age	Seizure type	Baseline Seizure frequency	Etiology	Epilepsy duration (yr)	Concomitant AEDs
1	M	42	Focal impaired awareness	4/mo	Infectious	2	OXC 450 mg
2	M	26	Focal to bilateral tonic-clonic	2/mo	Structural	20	OXC 1800 mg PGB 150 mg CZP 1.5 mg
3	M	56	Focal impaired awareness	1/yr	Infectious	0.5	OXC 1425 mg PGB 75 mg
4	M	35	Focal impaired awareness	2/yr	Unknown	7	OXC 900 mg
5	M	45	Focal aware	4/mo	Structural	1.5	OXC 600 mg PGB 300 mg CZP 1 mg
6	M	52	Focal impaired awareness	N/A	Structural	0.5	CZP 0.5 mg
7	M	27	Focal to bilateral tonic-clonic	1/yr	Structural	2	None
8	F	39	Generalized	1/mo	Unknown	4	None
9	M	34	Focal to bilateral tonic-clonic	N/A	Infectious	28	VPA 300 mg
10	F	41	Generalized	4/yr	Unknown	35	ZNS 100 mg
11	M	74	Focal impaired awareness	N/A	Unknown	7	ZNS 100 mg

No, number; M, male; F, female; mo, month; yr, year; AED, antiepileptic drug; OXC, oxcarbazepine; PGB, pregabalin; CZP, clonazepam; VPA, valproic acid; N/A, not available

**Table 2:** Manifested psychiatric adverse events and levetiracetam dosages at the point of the symptoms occurrence.

No	Manifestations	LEV dose (mg/day)
1	Aggression, Irritability	2000
2	Psychosis (auditory hallucination, delusion)	2000
3	Nervousness	1500
4	Aggression, Irritability	1500
5	Aggression, Irritability, Nervousness	1500
6	Aggression, Irritability	1000
7	Aggression, Irritability	1000
8	Psychosis (auditory hallucination)	1000
9	Aggression, Irritability, Nervousness	1000
10	Psychosis (auditory hallucination)	500
11	Aggression, Irritability	250

No, number; M, male; F, female; LEV, levetiracetam.

### Results of HLA genotyping

The results of the genotyping of the LEV-PAEs group patients are presented in Table 3. The genotype frequencies of the HLA genes in the LEV-PAEs group, the LEV-tolerant group and the general Korean population are shown in Table 4. The genotype frequency of the HLA-A*1101 allele was significantly higher in the LEV-PAEs group compared to both the LEV-tolerant group (p = 0.021, OR 4.80, 95% CI 1.30–17.74) and the general Korean population (p = 0.015, OR 4.62, 95% CI 1.38–15.45). On the other hand, the genotype frequency of the HLA-A*1101 allele was similar between the LEV-tolerant group and the general Korean population. Three HLA alleles, which have been reported to be associated with schizophrenia,[9, 10, 20, 21] are displayed in Table 4. These alleles were not significantly correlated with LEV-induced PAEs.

**Table 3:** Human leukocyte antigen genotype of the patients with levetiracetam-induced psychiatric adverse events.

No	HLA-A	HLA-B	HLA-C	HLA-DRB1	HLA-DQB1
1	0201/0206	3501/5401	0102/0303	1407/1501	0503/0602
2	1101/3303	1501/5801	0302/0401	0401/0406	0301/0302
3	1101/3101	1501/5102	0401/1502	0406/1501	0302/0602
4	0206/1101	3501/6701	0303/0702	0901/1101	0301/0303
5	1101/3303	1501/5101	0302/0401	0406/1301	0302/0603
6	2601/3001	1302/5401	0102/0602	0701/1405	0202/0503
7	2402/2402	1507/5101	0303/1402	0403/1501	0301/0302
8	0203/2402	3802/5401	0102/0702	0405/1502	0401/0501
9	1101/2402	0702/4002	0304/0702	0101/1406	0301/0501
10	0201/3004	1401/4001	0328/0802	0404/1101	0301/0402
11	1101/3303	3501/4403	0303/0706	0701/1405	0202/0503

No, number; HLA, human leukocyte antigen

**Table 4:** The frequencies of HLA genes in the LEV-PAE group, LEV-tolerant group and general Korean population, and the odds ratios among the three groups.

HLA allele	Frequency			LEV-PAE vs. LEV-tolerant		LEV-PAE vs. General		LEV-tolerant vs. General	
	LEV-PAE (%) (n = 11)	LEV-tolerant (%) (n = 80)	General population (%) (n = 485)	OR (95% CI)	p-value	OR (95% CI)	p-value	OR (95% CI)	p-value
HLA alleles most frequently identified in the LEV-PAE group									
A*1101	6 (54.55)	16 (20.00)	100 (20.62)	**4.80 (1.30–17.74)**	0.02*	**4.62 (1.38–15.45)**	0.02*	0.96 (0.53–1.74)	0.90
DQB1*0301	5 (45.45)	23 (28.75)	121 (24.95)	1.77 (0.51–6.15)	0.50	2.15 (0.67–6.90)	0.19	1.21 (0.72–2.05)	0.47
Cw*0303	4 (36.36)	19 (23.75)	110 (22.68)	1.84 (0.49–6.95)	0.46	1.95 (0.56–6.78)	0.29	1.06 (0.61–1.85)	0.83
DQB1*0302	4 (36.36)	21 (26.25)	97 (20.00)	2.01 (0.57–7.01)	0.31	2.86 (0.89–9.20)	0.08	1.42 (0.83–2.46)	0.20
HLA alleles previously reported to be associated with schizophrenia									
DRB1*0101	1 (9.09)	8 (10.00)	64 (13.20)	0.90 (0.10–7.97)	1.00	0.66 (0.08–5.23)	1.00	0.73 (0.34–1.59)	0.47
DQB1*0303	1 (9.09)	22 (27.50)	104 (21.44)	0.26 (0.03–2.18)	0.28	0.37 (0.05–2.90)	0.47	1.39 (0.81–2.38)	0.23
DQB1*0602	2 (18.18)	8 (10.00)	67 (13.81)	2.00 (0.37–10.92)	0.35	1.39 (0.29–6.56)	0.66	0.69 (0.32–1.50)	0.35

HLA, human leukocyte antigen; LEV-PAE, levetiracetam-induced psychiatric adverse event; OR, odds ratio

* p-Value <0.05

### In silico analysis and molecular docking of levetiracetam

**Fig 1:**
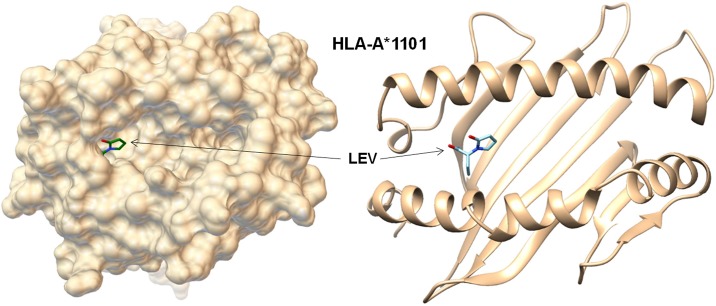
In-silico modeling of the molecular interaction between HLA-A*1101 and LEV. The LEV molecule was predicted to be docked into the P1 pocket of HLA-A*1101 with relatively higher affinity (5.1 kcal/mol) compared to other HLA-A subtypes. HLA, human leukocyte antigen; LEV, levetiracetam.

The binding affinity of the LEV molecule to HLA-A*1101 was compared to the binding affinity obtained from docking with seven HLA-A alleles whose crystallographic structures are available. The LEV molecule was predicted to be docked into the P1 pocket of HLA-A*1101 (Fig 1) with a docking score of 5.1 kcal/mol, which was the second highest affinity in those with the eight HLA-A alleles ranging from 4.8 to 5.2 kcal/mol.

## Discussion

This is the first study to investigate the HLA-related risk factors for LEV-induced PAEs in epilepsy patients. We identified significant associations between LEV-induced PAEs and the HLA-A*1101 allele. The in-silico analysis revealed that the LEV molecule was predicted to dock into the P1 pocket of the MHC class I heterodimer including A*1101. This finding provides a new insight into the pathogenesis of LEV-induced PAEs, and further studies using larger numbers of patients are required.

LEV is a second-generation antiepileptic drug that has been proven to be effective in focal and generalized epilepsy,[1–3] and is widely used due to its relatively few drug interactions and favorable tolerability.[4, 5] However, PAEs are reported to occur in 3.8–10.1% of patients taking LEV [1, 7, 22–24] and may even result in suicide in extreme cases.[25] Almost 7% of patients discontinue LEV due to PAEs, which have proven to be the most common reason for withdrawal in patients taking LEV.[26] Unfortunately, the pathophysiology of LEV-induced PAEs has remained unclear until now.

In recent studies which investigated the possible associations among the location of brain lesion, the use of AEDs and the development of PAEs in patients with brain tumor-related epilepsy, frontal lobe tumors were highly associated with the development of PAEs after LEV treatment.[27, 28] In addition, LEV-induced PAEs were more commonly reported in specific populations who had previous history of febrile convulsion or status epilepticus, previous history of psychiatric disorders, and familial history of psychiatric disorders.[7, 29]

PAEs of LEV occur unexpectedly and independently of the dose, occurring even at doses below 1000 mg/day,[30] which suggests that this adverse event is an idiosyncratic adverse drug reaction. Idiosyncratic adverse drug reactions of AEDs are unpredictable and are thought to have an underlying genetic etiology.[15, 16, 31] However, the genetic risk factors of LEV-induced PAEs have not been thoroughly investigated; thus, the occurrence of PAEs can only be detected by LEV administration to every patient. Helmstaedter et al. conducted multiple SNP analyses of genes related to dopaminergic activity in 398 patients with epilepsy and taking LEV.[32] They revealed a higher load of adverse psychotropic side effects of LEV in patients carrying rs1800497 (dopamine receptor D2-associated ANKK1 TAQ-1A), which is associated with decreased dopaminergic activity. However, none of the HLA-related risk factors for LEV-induced PAEs have yet been identified.

In this study, the frequency of the HLA-A*1101 allele was significantly higher in the LEV-PAE group than in the LEV-tolerant group or the general Korean population, which suggests that LEV-induced PAEs may also be related to the HLA genes. Previous studies have shown that immune responses are involved in the development of psychiatric diseases, including schizophrenia and bipolar disorders, and have suggested several HLA genes related to the pathogenesis of these diseases. Two previous studies have shown that patients with schizophrenia had higher rates of the HLA-DRB1*0101 genes than the controls in the Japanese population.[9, 21] In addition, the frequency of HLA-DQB1*0303 had a positive association with schizophrenia, whereas HLA-DQB1*0602 had a negative association in the Chinese population.[10] Two additional studies suggest a negative association between HLA-DQB1*0602 and schizophrenia in the African-American population.[20, 33] Patients with bipolar disorder showed significantly increased allele frequencies of the HLA-A29 and HLA-B21 antigens compared to the controls in the Spanish population.[11] Similarly, HLA-A29 and HLA-B54 were detected more frequently, while HLA-B51 and HLA-DRB1*02 were less frequent in patients with bipolar disorder in a Korean population.[34]

HLA-A*1101 is the dominant serotype of HLA-A11 (A11), which is one of the most common HLA class I genotypes in the world.[35] HLA-A forms a receptor structure in the human MHC class I molecules, which present antigenic peptides to CD8+ T cells and trigger the cytotoxic T lymphocyte (CTL) response.[36] Many transgenic HLA mice are used as models of the human immune responses. HLA-A*1101 transgenic mice, which contain the human HLA-A11 molecule, are well-known models for studying MHC Class I antigen presentation. Therefore, HLA-A*1101 transgenic mice are suitable for the investigation of human immune reactions to viral infections and are thus widely used for vaccine development studies.[35, 37–39]

LEV-induced idiosyncratic reactions may be caused by directly triggering the CTL response via the HLA system. The in-silico docking analysis has demonstrated that the LEV molecule binds within the P1 pocket of the peptide binding groove of HLA-A*1101, with a relatively stronger affinity than other HLA subtypes. Several reports have shown that drug molecules can directly interact with HLA molecules in combination with specific peptides and trigger unexpected immune reactions.[40] Abacavir can bind within the F pocket of the peptide-binding groove of HLA-B*57:01 and induce CTL responses.[41] Likewise, carbamazepine can be loaded in the B pocket of the HLA-B*1502 molecule and activate CTLs without the involvement of intracellular drug metabolism or antigen processing.[42] Although the detailed mechanism of LEV-induced idiosyncratic reactions needs to be investigated further, our data suggest that LEV-induced PAEs occur as an idiosyncratic reaction via the HLA system.

One of the limitations of our study is the small number of patients included in the LEV-PAE group. Additionally, an objective psychiatric assessment scale was not used to evaluate the PAEs, which were mainly assessed by the clinician’s judgement. Therefore, we have excluded all the patients with unclear symptoms or unclear causality with LEV administration from the larger number of patients suspected to have PAEs. Despite the reduction in the number of patients, we made great efforts to select definite LEV-induced PAEs, which were supported by the spontaneous improvement of the psychiatric symptoms after drug removal.

In conclusion, we suggest for the first time that the HLA system is associated with LEV-induced PAEs, and the HLA-A*1101 allele could be a risk factor for the development of psychiatric symptoms. Identifying the genetic risk factors for LEV-induced PAEs, along with other known risk factors, will be significantly beneficial to clinicians when prescribing LEV. To strengthen the findings obtained from the current study, additional studies using objective assessment instruments of psychiatric symptoms in larger numbers of patients or in different ethnic groups will be required in the near future.
